# Sequential Logic-Gated Prioritization of microRNA Networks Identifies DKK1 as a Candidate Associated With Metabolic Dysfunction-Associated Steatotic Liver Disease-Hepatocellular Carcinoma (MASLD-HCC) and Metastasis

**DOI:** 10.7759/cureus.111652

**Published:** 2026-06-28

**Authors:** Tyler L Bissoondial, Prakash Narayan

**Affiliations:** 1 Pathology and Laboratory Medicine, University of North Carolina at Chapel Hill, Chapel Hill, USA; 2 Bioinformatics, Nodes and Edges LLC, Raleigh, USA

**Keywords:** cirrhosis, dkk1, fibrosis, hepatocellular carcinoma, liver, masld, metastasis, microrna, oncogene

## Abstract

Metabolic dysfunction-associated steatotic liver disease (MASLD) is projected to become the leading cause of hepatocellular carcinoma (HCC) worldwide. Unlike fibrosis in other organs, liver fibrosis carries a higher risk of malignant transformation, suggesting that hepatic microenvironmental signaling promotes oncogenesis and metastasis. Our overarching hypothesis is that MASLD-HCC and metastasis are associated with reduced expression of tumor-suppressive microRNAs and increased expression of oncogenes. We undertook an in silico, logic-gated, prioritization strategy integrating MASLD-HCC-associated microRNA networks, HCC biomarker-associated microRNA networks, tumor suppressor scoring, and expression analyses to identify candidates associated with MASLD-HCC and metastasis. Sequential Boolean AND logic gating identified hsa-miR-101-3p, -206, and -613, each with tumor-suppressive activity and reduced expression in HCC and other cancers. All three microRNAs converged on a single node, Dickkopf-related protein 1(*dkk1*)*. *Previous studies have associated DKK1 with migration, invasion, and angiogenesis, promoting tumor aggressiveness, vascular invasion, pulmonary and lymphatic metastasis, recurrence, and poor overall survival. Integration in silico of network biology, transcriptomic analysis, and literature-derived mechanistic evidence supports a hypothesis for a loss-of-control model in which MASLD-HCC is associated with reduced expression of tumor-suppressive microRNAs and increased expression of oncogenic DKK1.

## Introduction

Metabolic dysfunction-associated steatotic liver disease (MASLD) affects ~100 million adults in the United States alone and ~1.3 billion adults worldwide [1]. Driven in large part by diabetes, obesity, and metabolic syndrome epidemics, MASLD starts with simple steatosis or the accumulation of lipid droplets within the liver. When inflammation follows, the condition is termed metabolic dysfunction-associated steatohepatitis (MASH), a stage accompanied by an increase in liver enzymes [2]. The continuum can progress to MASH with fibrosis, and then cirrhosis, which is severe scarring of the liver, disfigurement of the organ, and potential for declining liver function [1,2]. Cirrhosis not only represents a risk for liver failure but also poses a significant risk for progression to hepatocellular carcinoma (HCC). Indeed, 80%-90% of HCC develops in cirrhotic livers, albeit not all related to MASLD [1]. In so far as MASLD-HCC is concerned, clinical data indicate that 20%-30% of such cases occur in the absence of cirrhosis [1]. Given the sheer size of the metabolic disease epidemic, MASLD is projected to be the leading cause of HCC, with or without cirrhosis, within the next few decades [1].

Failed and/or maladaptive tissue repair and an epithelial-to-mesenchymal transition (EMT) represent fibrotic disease-associated mechanisms shared across multiple organs, including the liver, kidney, lung, and heart [3]. Nevertheless, fibrosis in these organs does not carry a risk of malignancy that is comparable to the risk for HCC from MASLD. These statistics suggest that factors associated with the hepatic microenvironment drive MASLD toward HCC. Both experimental [2] and clinical [4] findings inform multiple circulating biomarkers that support a diagnosis of HCC. These biomarkers include alpha fetoprotein (AFP), des γ carboxyprothrombin (DCP/PIVKA II), glypican-3, osteopontin (OPN), Golgi protein-73 (GP73), and Dickkopf-related protein 1 (DKK1) [2]. Expression levels of these biomarkers are increased in HCC [2,4], driven by increased expression of their mRNA, which in turn is driven by reduced expression levels of microRNAs regulating these mRNAs [5]. Many, if not all, of these biomarkers are also oncogenic, promoting primary tumor formation and/or metastasis [6,7]. Our overarching hypothesis is that MASLD-HCC and metastasis are associated with reduced expression of tumor-suppressive microRNAs and increased expression of oncogenes. We undertook an in silico, sequential, logic-gated prioritization of microRNA networks toward uncovering candidate(s) associated with MASLD-HCC and metastasis.

## Materials and methods

This in silico study integrates publicly available transcriptomic datasets with external network-based target prediction resources and literature-curated functional evidence to prioritize candidate microRNA-mRNA regulatory axes associated with MASLD-HCC and metastasis. The approach employs a sequential, logic-gated strategy comprising four Boolean AND gates (Table 1) with advancement of those candidates meeting the prespecified criteria. The first gate selects for microRNAs that belong to both the MASLD-HCC network (miRNet) [8] and HCC biomarker network (miRDB) [9]. The next gate selects for microRNAs that are also tumor-suppressive, with the third gate allowing for microRNAs whose expression levels are downregulated in HCC and other cancers. The fourth gate selects microRNAs that regulate mRNA with oncogenic and metastatic activity. Since no established scoring framework exists for integrated microRNA prioritization in MASLD-HCC, for the purpose of this exploratory study, an investigator-defined semi-quantitative rubric was developed based on principles of reproducibility, orthogonal validation, and cross-cohort concordance.

**Table 1:** Sequential logic-gated prioritization in silico to identify candidates with oncogenic and metastatic activity that are associated with MASLD-HCC and metastasis. MASLD-HCC: metabolic dysfunction-associated steatotic liver disease-hepatocellular carcinoma

Sequential logic-gated prioritization								
1	microRNA	MASLD-HCC network	AND	HCC biomarkers network				
2	microRNA	MASLD-HCC network	AND	HCC biomarkers network	AND	tumor suppressive		
3	microRNA	MASLD-HCC network	AND	HCC biomarkers network	AND	tumor suppressive	AND	downregulated in HCC and other cancers
4	microRNA	MASLD-HCC network	AND	HCC biomarkers network	AND	tumor suppressive	AND	downregulated in HCC and other cancers

Bioinformatics query

A MASLD-HCC interactome was constructed using miRNet [8] by querying the microRNA-disease module using the in-built terms “fatty-liver, Non-alcoholic”, non-alcoholic-fatty liver”, “non-alcoholic Fatty Liver Disease”, “Metabolic-Associated Fatty Liver Disease”, “Liver Fibrosis”, “Liver Cirrhosis”, “Liver Neoplasms”, and “Carcinoma Hepatocellular”. A list of microRNAs regulating HCC biomarkers [2,5-7] afp (AFP), f2 (prothrombin), spp1 (OPN), gpc3 (glypican-3), golm1 (GP73), and dkk1 (DKK1) was generated using miRDB [9].

Scoring rubric for confidence in the tumor-suppressive nature of the microRNA

The published literature (PubMed) and The Cancer Genome Atlas (TCGA)-Liver Hepatocellular Carcinoma (LIHC) data collection [10] were queried using the terms “miR-101-3p”, “-206” and “-613”, “LIHC” or “HCC”, “tumor”, “cancer”, and/or “suppress” to determine whether or not a microRNA has tumor suppressor activity against HCC and other cancers. A tumor suppressor score was assigned using a semi-quantitative five-dimensional rubric (0-3 points each; maximum score = 15; Appendices A, B) [11]. For the purpose of this exploratory study, a microRNA with a total score > 12 was deemed tumor-suppressive.

Scoring rubric for confidence in the downregulation of microRNA in HCC and other cancers 

The published literature (PubMed) and TCGA-LIHC and Gene Expression Omnibus (GEO) [10] were queried using the terms “miR-101-3p”, “-206” and “-613”, “LIHC” or “HCC”, “tumor”, “cancer”, and/or “expression” to determine whether or not expression of the microRNA is downregulated in HCC and other cancers using a semi-quantitative five-dimensional rubric (maximum score = 100; Appendices C, D) [11]. For the purpose of this exploratory study, a microRNA with a total score > 80 was deemed to be downregulated in HCC and cancers.

## Results

**Figure 1:**
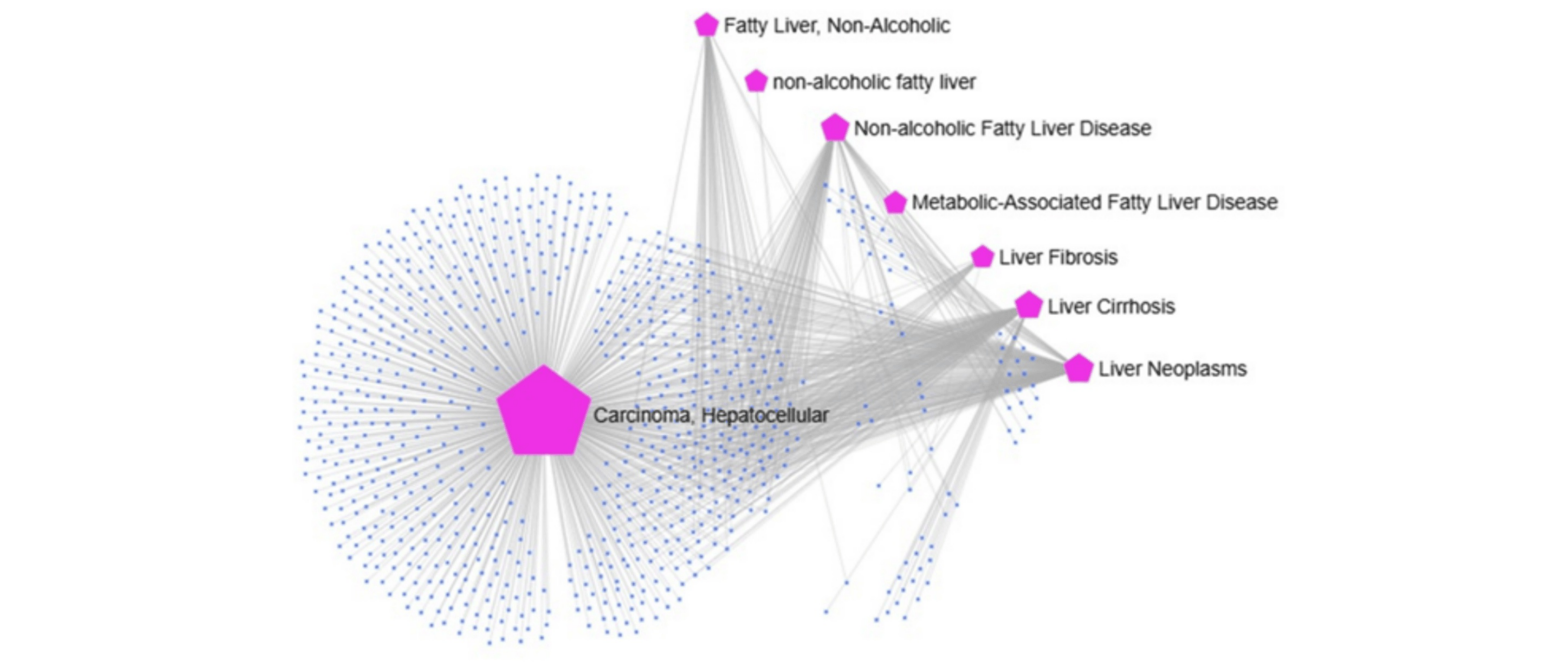
The MASLD-HCC network. Forced atlas representation of the MASLD-HCC network. Blue nodes represent microRNA, pink nodes represent mRNA, and gray lines represent interactions between the two. MASLD-HCC: metabolic dysfunction-associated steatotic liver disease-hepatocellular carcinoma

Our overarching hypothesis is that MASLD-HCC and metastasis are associated with reduced expression of tumor-suppressive microRNAs that regulate HCC oncogenes. We sought to first identify candidate microRNAs by constructing a MASLD-HCC network. This network encompasses microRNAs regulating the MASLD-HCC continuum, viz., steatosis, steatosis + inflammation, fibrosis, cirrhosis, and cancer. As seen in Figure 1, multiple microRNAs are involved in more than one disease stage. The microRNAs constituting the MASLD-HCC network are found in Appendix E.

Expression levels of biomarkers AFP, prothrombin/DCP/PIVKA II, glypican-3, OPN, GP73, and DKK1 are elevated in HCC [2,4]. Not only are these biomarkers diagnostic of HCC, but many, if not all, are oncogenic [6,7]. Using miRDB, we identified microRNAs associated with the HCC biomarker network (Appendix F).

Next, we gated for microRNAs residing within the MASLD-HCC AND HCC biomarker networks. These nine microRNAs and their target mRNA are listed in Table 2.

**Table 2:** microRNAs within the MASLD-HCC AND HCC biomarker network. spp1: secreted phosphoprotein 1 (osteopontin or OPN); golm1: Golgi phosphoprotein 73 (GP73); dkk1: Dickkopf-related protein 1; MASLD-HCC: metabolic dysfunction-associated steatotic liver disease-hepatocellular carcinoma

microRNA	HCC biomarker gene
hsa-miR-577	spp1
hsa-miR-130b-5p	spp1
hsa-miR-9500	golm1
hsa-miR-101-3p	dkk1
hsa-miR-206	dkk1
hsa-miR-613	dkk1
hsa-miR-103a-3p	dkk1
hsa-miR-107	dkk1
hsa-miR-219a-1-3p	dkk1

To identify microRNAs residing within the MASLD-HCC AND HCC biomarker network AND having tumor-suppressive activity, we queried the published literature and large cancer repositories. Tumor-suppressive scores for the nine microRNAs from Table 1 are listed in Appendix G. Those microRNAs with scores satisfying the prespecified threshold for tumor suppression, together with the mechanism of action, are listed in Table 3. Interestingly, all three microRNAs, viz., hsa-miRs 101-3p, -206, and -613, converge on *dkk1*.

**Table 3:** microRNAs within the MASLD-HCC AND HCC biomarker network AND tumor suppressive together with the purported mechanism(s) of action and their target HCC biomarker mRNA. EZH2: enhancer of zeste homolog 2; SOX9: SRY-box transcription factor 9; dkk1: Dickkopf-related protein 1; MASLD-HCC: metabolic dysfunction-associated steatotic liver disease-hepatocellular carcinoma; EMT: epithelial-to-mesenchymal transition

microRNA	Tumor suppressor confidence score	Effect	Target	HCC biomarker gene
hsa-miR-101-3p	14	Inhibits proliferation, suppresses EMT & metastasis, promotes apoptosis	EZH2 [10,12,13]	dkk1
hsa-miR-206	13	Inhibits proliferation, inhibits motility and invasion, inhibits survival signaling	c-Met PI3/AKT [10,14,15]	dkk1
hsa-miR-613	12	Inhibits proliferation, suppresses migration/invasion	SOX9 [10,16-18]	dkk1

When we queried for microRNAs residing within the MASLD-HCC AND HCC biomarker networks AND are tumor-suppressive AND downregulated in HCC and other cancers, hsa-miRs 101-3p, -206, and -613 met these criteria (Table 4). Listed in Appendix H are the component scores making up their total score.

**Table 4:** microRNAs within the MASLD-HCC AND HCC biomarker network AND tumor suppressive AND downregulated in HCC and other cancers. dkk1: Dickkopf-related protein 1; MASLD-HCC: metabolic dysfunction-associated steatotic liver disease-hepatocellular carcinoma

microRNA	Downregulated in tumors, confidence score	HCC biomarker gene
hsa-miR-101-3p [10,19]	94	dkk1
hsa-miR-206 [10,20-22]	90	dkk1
hsa-miR-613 [10,23]	85	dkk1

Since sequential use of Boolean AND logic gates converged on dkk1, we queried whether DKK1 has oncogenic and metastatic activity. Previously published independent observations attest to the oncogenic and metastatic activity of DKK1. Huang et al. [24] reported elevated DKK1 expression in aggressive HCC tissues and demonstrated its association with increased invasion and metastatic recurrence. Mechanistically, activation of the DKK1-CKAP4 signaling axis has been shown to promote an aggressive HCC phenotype [25], while other studies have linked DKK1 overexpression to increased migration and invasion and as an independent prognostic indicator for overall survival and cumulative recurrence in patients with HCC through pathways including β-catenin/MMP7 signaling [26,27]. DKK1 has also been reported to enhance proliferation, migration, and metastatic dissemination, including lung metastasis, through activation of downstream pathways such as Akt/NUAK1 signaling [28]. DKK1-mediated modulation of the tumor microenvironment through inflammatory and TGFβ1-associated mechanisms may further facilitate invasive behavior [29]. Collectively, these findings indicate that DKK1 functions beyond a biomarker of HCC, acting as an oncogenic candidate that promotes tumor aggressiveness, invasion, and metastasis. Finally, although DKK1 levels are not routinely measured, both experimental and clinical studies have reported increased expression of this biomarker in HCC [2,30].

## Discussion

In this study, we applied a sequential Boolean AND logic-gated prioritization strategy integrating MASLD-HCC microRNA networks, HCC biomarker microRNA networks, tumor suppressor scoring, and tumor expression analyses to uncover an oncogenic candidate associated with MASLD-HCC and metastasis. This approach identified hsa-miR-101-3p, hsa-miR-206, and hsa-miR-613, tumor-suppressive microRNAs downregulated in HCC and other cancers converging on oncogenic and metastatic DKK1. These findings support a hypothesis for a loss-of-control model in which MASLD-HCC and metastasis are associated with reduced expression of tumor-suppressive microRNAs and increased expression of DKK1.

A central observation emerging from this work, albeit in silico in nature, is the convergence of multiple independent tumor-suppressive microRNAs on DKK1. Although each of these microRNAs has previously been implicated in tumor suppression through distinct mechanisms [10,12-18], our network-based analysis suggests that their coordinated loss may converge functionally on a shared oncogenic axis. hsa-miR-101-3p has been associated with suppression of proliferation, EMT, and invasion in HCC [10,12,13,20]. hsa-miR-206 similarly suppresses migratory and invasive phenotypes [10,14,15,20-22], whereas hsa-miR-613 has been linked to inhibition of proliferation and metastatic behavior [10,16-18,23]. The observation that all three microRNAs target dkk1 suggests that DKK1 may represent a common denominator for these signaling pathways. Indeed, previously published reports support a functional role for DKK1 in HCC progression beyond its role as a biomarker. Elevated DKK1 expression has been associated with aggressive tumor behavior, including enhanced proliferation, invasion, metastatic recurrence, and poor clinical outcomes. Mechanistic studies implicate DKK1-dependent signaling networks, including CKAP4, β-catenin/MMP7, Akt/NUAK1, and TGFβ1-dependent tumor microenvironmental pathways, in promoting HCC aggressiveness. These data suggest that DKK1 may be more than a bystander in metabolically driven HCC and metastasis.

The sequential Boolean logic-gated prioritization strategy used here offers several conceptual advantages. This approach integrates disease association, biomarker linkage, tumor-suppressive function, and downregulation status, enriching biologically meaningful candidates. This methodology may be particularly useful in complex multifactorial diseases such as MASLD-HCC, where extensive molecular heterogeneity complicates the identification of actionable drivers. Notably, this approach identified a relatively small cohort of microRNAs from a much larger network, suggesting that convergence-based filtering may help prioritize therapeutically relevant regulatory axes. These findings may also have translational implications. First, the combined loss of hsa-miR-101-3p, hsa-miR-206, and hsa-miR-613 together with elevated DKK1 expression may serve as a molecular signature associated with aggressive MASLD-HCC. Such a signature could potentially improve risk stratification beyond conventional biomarkers alone. Second, restoration of tumor-suppressive microRNAs or direct targeting of DKK1 signaling pathways may represent therapeutic opportunities. Given that DKK1 is secreted and detectable in circulation, it may also serve as a theragnostic target. Finally, because DKK1 appears to regulate metastatic pathways, inhibition of DKK1 could potentially suppress both primary tumor progression and metastasis.

**Figure 2:**
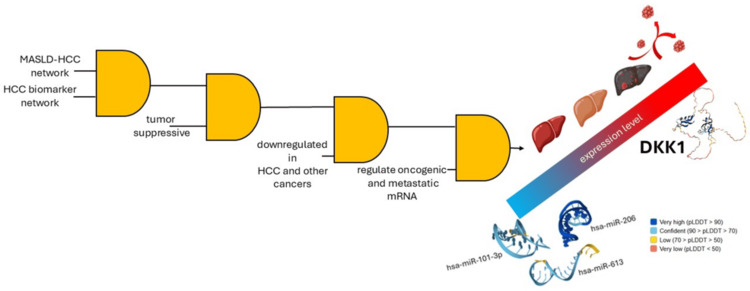
Sequential logic-gated prioritization of microRNA networks. In silico Boolean sieving converges on DKK1 as an oncogenic and metastatic candidate in MASLD-HCC. Figure created by the authors using Microsoft PowerPoint (Microsoft Corp., Redmond, WA, USA) MASLD-HCC: metabolic dysfunction-associated steatotic liver disease-hepatocellular carcinoma

Several limitations should be acknowledged. First, the present study is bioinformatic- and literature-based, and therefore, causal relationships remain inferential. Experimental validation of direct microRNA-DKK1 regulatory interactions in MASLD-HCC models will be important. Second, although the scoring rubrics used to classify tumor suppressive activity and downregulation were designed to provide semi-quantitative rigor, such systems inherently involve subjective weighting. Third, the confidence thresholds employed to advance microRNA candidates are investigator-defined. Fourth, HCC is molecularly heterogeneous, and the contribution of the microRNA-DKK1 axis may vary across etiologies and tumor subtypes. Future analyses comparing MASLD-associated HCC with viral- and alcohol-associated HCC will be necessary to determine etiologic specificity. Future studies incorporating transcriptomic, proteomic, and spatial analyses of MASLD-HCC tissues will be necessary to clarify the temporal and spatial dynamics of this regulatory network. Despite these limitations, sequential, logic-gated prioritization of microRNA networks demonstrates a loss-of-control model with DKK1 as a candidate associated with MASLD-HCC and metastasis (Figure 2).

## Conclusions

Integration of bioinformatic and literature-derived evidence, coupled with logic-gated sieving, supports a hypothesis for a loss-of-control model in which tumor-suppressive hsa-miRs-101-3p, -206, and -613 are downregulated. Furthermore, these microRNAs converge on DKK1, which has previously been reported to harbor oncogenic and metastatic activity. These findings generate a testable hypothesis that DKK1 signaling is a potentially important mechanistic and translational pathway in MASLD-HCC and metastasis. Additional laboratory and clinical experiments will be needed to confirm whether reduced levels of these microRNAs directly cause increased DKK1 activity and whether this, in turn, drives MASLD-HCC and metastasis.

## Appendices

Appendix A 

**Table 5:** Rubric for determining the tumor-suppressive nature of the microRNA. HCC: hepatocellular carcinoma

Dimension	Score = 0	Score = 3
Expression consistency in HCC and other cancers	Inconsistent expression across studies	Consistently downregulated
Functional validation in HCC and other cancers	No functional validation	Effects of under-/overexpression
Mechanistic clarity in HCC and other cancers	Mechanism unknown	Validated targets with coherent pathway-level evidence
Clinical correlation in HCC and other cancers	No clinical association	Significant correlation with tumor stage and/or survival
Oncogenic safety in HCC and other cancers	Evidence of oncogenic activity	Clean tumor suppressor profile

Appendix B 

**Table 6:** Confidence thresholds for the tumor-suppressive nature of the microRNA.

Score	Interpretation
14-15	Very high confidence
12-13	Strong confidence
10-11	Moderate confidence
7-9	Suggestive
0-6	Weak/insufficient evidence

Appendix C

**Table 7:** Rubric for determining if the expression level of a microRNA is downregulated in HCC and other cancers. HCC: hepatocellular carcinoma; GEO: Gene Expression Omnibus

Dimension	Evidence	Score
Reduced expression in HCC and other cancers	≥6 independent studies	28-30
	4-5 studies	24-27
	2-3 studies	18-23
	1 study	10-17
	No direct human data	<9
Sample size HCC and cancers + control	>500	18-20
	250-500	15-17
	100-249	12-14
	50-99	8-11
	<50	<7
Functional validation in HCC and other cancers	In vitro + in vivo (xenograft) + multiple validated targets in cancers	18-20
	Robust in vitro evidence (proliferation, invasion, apoptosis)	14-17
	Limited functional assays	8-13
	Predicted targets only	1-7
	No functional data	0
Clinical correlation in HCC and other cancers	Survival + stage + invasion/metastasis associations	13-15
	Any 2 of the above	10-12
	One clinical association	6-9
	Weak or non-significant trends	1-5
	None	0
Large dataset validation in HCC and other cancers	PubMed, The Cancer Genome Atlas (TCGA) + ≥1 GEO datasets confirm downregulation	13-15
	TCGA only or multiple GEO datasets	10-12
	Single dataset support	6-9
	Inconsistent datasets	1-5
	No dataset support	0

Appendix D

**Table 8:** Confidence thresholds for downregulation of microRNA expression level in HCC and other cancers. HCC: hepatocellular carcinoma

Score	Interpretation
90-100	Very high confidence
80-89	Strong confidence
70-79	Moderate confidence
60-69	Suggestive
0-59	Weak/insufficient evidence

Appendix E 

**Table 9:** microRNAs within the MASLD-HCC network. MASLD-HCC: metabolic dysfunction-associated steatotic liver disease-hepatocellular carcinoma

microRNAs								
hsa-let-7a-1	hsa-miR-129-1	hsa-miR-133a-2	hsa-miR-363	hsa-miR-193b	hsa-miR-33a-5p	hsa-miR-942	hsa-miR-498	hsa-miR-5
hsa-let-7a-2	hsa-miR-148a	hsa-miR-135a-1	hsa-miR-365a	hsa-miR-497	hsa-miR-92a-3p	hsa-miR-506	hsa-miR-4488	hsa-miR-6499
hsa-let-7a-3	hsa-miR-30d	hsa-miR-135a-2	hsa-miR-302b	hsa-miR-181d	hsa-miR-101-3p	hsa-miR-129	hsa-miR-3677	hsa-miR-4715
hsa-let-7b	hsa-miR-139	hsa-miR-140	hsa-miR-376c	hsa-miR-525	hsa-miR-29b-3p	hsa-miR-320d-1	hsa-miR-4746	hsa-miR-6766
hsa-let-7c	hsa-miR-10a	hsa-miR-141	hsa-miR-369	hsa-miR-524	hsa-miR-103a-3p	hsa-miR-34	hsa-miR-181a	hsa-miR-467b
hsa-let-7d	hsa-miR-10b	hsa-miR-142	hsa-miR-370	hsa-miR-519a-1	hsa-miR-106a-5p	hsa-miR-1266	hsa-miR-218	hsa-miR-40
hsa-let-7e	hsa-miR-34a	hsa-miR-143	hsa-miR-372	hsa-miR-500a	hsa-miR-107	hsa-miR-199	hsa-miR-1	hsa-miR-44
hsa-let-7f-1	hsa-miR-181a-2	hsa-miR-144	hsa-miR-373	hsa-miR-503	hsa-miR-198	hsa-miR-6838	hsa-miR-125b	hsa-miR-466g
hsa-let-7f-2	hsa-miR-181b-1	hsa-miR-145	hsa-miR-374a	hsa-miR-513a-1	hsa-miR-199a-5p	hsa-miR-124a	hsa-miR-194	
hsa-miR-15a	hsa-miR-181c	hsa-miR-152	hsa-miR-377	hsa-miR-513a-2	hsa-miR-199a-3p	hsa-miR-130	hsa-miR-138	
hsa-miR-16-1	hsa-miR-182	hsa-miR-191	hsa-miR-378a	hsa-miR-532	hsa-miR-182	hsa-miR-196	hsa-miR-16	
hsa-miR-17	hsa-miR-183	hsa-miR-9-1	hsa-miR-379	hsa-miR-455	hsa-miR-137	hsa-miR-209	hsa-miR-92a	
hsa-miR-18a	hsa-miR-187	hsa-miR-9-2	hsa-miR-380	hsa-miR-92b	hsa-miR-9	hsa-miR-922	hsa-miR-29b	
hsa-miR-19a	hsa-miR-196a-2	hsa-miR-125a	hsa-miR-381	hsa-miR-574	hsa-miR-126*	hsa-miR-378b	hsa-miR-101	
hsa-miR-20a	hsa-miR-199a-2	hsa-miR-125b-2	hsa-miR-382	hsa-miR-576	hsa-miR-184	hsa-miR-378e	hsa-miR-124	
hsa-miR-21	hsa-miR-199b	hsa-miR-126	hsa-miR-383	hsa-miR-589	hsa-miR-206	hsa-miR-181	hsa-miR-105	
hsa-miR-22	hsa-miR-203a	hsa-miR-127	hsa-miR-340	hsa-miR-590	hsa-miR-375	hsa-miR-4449	hsa-miR-7	
hsa-miR-23a	hsa-miR-204	hsa-miR-134	hsa-miR-330	hsa-miR-615	hsa-miR-326	hsa-miR-484	hsa-miR-103a	
hsa-miR-24-1	hsa-miR-205	hsa-miR-136	hsa-miR-328	hsa-miR-625	hsa-miR-324-5p	hsa-miR-1273g	hsa-miR-26a	
hsa-miR-24-2	hsa-miR-210	hsa-miR-146a	hsa-miR-342	hsa-miR-627	hsa-miR-133b	hsa-miR-675	hsa-miR-24	
hsa-miR-25	hsa-miR-211	hsa-miR-149	hsa-miR-337	hsa-miR-629	hsa-miR-429	hsa-miR-1271	hsa-miR-19b	
hsa-miR-26a-1	hsa-miR-212	hsa-miR-150	hsa-miR-151a	hsa-miR-33b	hsa-miR-449a	hsa-miR-1303	hsa-miR-196a	
hsa-miR-26b	hsa-miR-181a-1	hsa-miR-154	hsa-miR-135b	hsa-miR-652	hsa-miR-451a	hsa-miR-320e	hsa-miR-135a	
hsa-miR-27a	hsa-miR-214	hsa-miR-185	hsa-miR-148b	hsa-miR-449b	hsa-miR-612	hsa-miR-618	hsa-miR-181b	
hsa-miR-28	hsa-miR-215	hsa-miR-186	hsa-miR-331	hsa-miR-411	hsa-miR-219a-1-3p	hsa-miR-421	hsa-miR-9	
hsa-miR-29a	hsa-miR-216a	hsa-miR-188	hsa-miR-324	hsa-miR-654	hsa-miR-130b	hsa-miR-1180	hsa-let-7f	
hsa-miR-30a	hsa-miR-218-1	hsa-miR-190a	hsa-miR-338	hsa-miR-660	hsa-miR-130b-5p	hsa-miR-613	hsa-miR-153	
hsa-miR-31	hsa-miR-219-1	hsa-miR-193a	hsa-miR-339	hsa-miR-542	hsa-miR-500a-5p	hsa-miR-650	hsa-miR-667	
hsa-miR-32	hsa-miR-219a-1	hsa-miR-194-1	hsa-miR-335	hsa-miR-767	hsa-miR-665	hsa-miR-1228	hsa-miR-103	
hsa-miR-33a	hsa-miR-221	hsa-miR-195	hsa-miR-345	hsa-miR-1224	hsa-miR-106	hsa-miR-1290	hsa-miR-1205	
hsa-miR-92a-2	hsa-miR-222	hsa-miR-200c	hsa-miR-196b	hsa-miR-1296	hsa-miR-486-1	hsa-miR-27	hsa-miR-44352	
hsa-miR-93	hsa-miR-223	hsa-miR-155	hsa-miR-423	hsa-miR-454	hsa-miR-217	hsa-miR-577	hsa-miR-6888	
hsa-miR-95	hsa-miR-224	hsa-miR-181b-2	hsa-miR-424	hsa-miR-320b-2	hsa-miR-1297	hsa-miR-320c-1	hsa-miR-4691	
hsa-miR-96	hsa-miR-200b	hsa-miR-128b	hsa-miR-425	hsa-miR-874	hsa-miR-30	hsa-miR-543	hsa-miR-6512	
hsa-miR-98	hsa-let-7g	hsa-miR-194-2	hsa-miR-18b	hsa-miR-708	hsa-miR-638	hsa-miR-122a	hsa-miR-1291	
hsa-miR-99a	hsa-let-7i	hsa-miR-106b	hsa-miR-20b	hsa-miR-744	hsa-miR-199a	hsa-miR-548f	hsa-miR-3118	
hsa-miR-100	hsa-miR-1-2	hsa-miR-29c	hsa-miR-431	hsa-miR-873	hsa-miR-371a	hsa-miR-466	hsa-miR-4730	
hsa-miR-101-1	hsa-miR-15b	hsa-miR-30c-1	hsa-miR-433	hsa-miR-1249	hsa-miR-513c	hsa-miR-350	hsa-miR-291	
hsa-miR-29b-1	hsa-miR-23b	hsa-miR-200a	hsa-miR-409	hsa-miR-1307	hsa-miR-571	hsa-miR-384	hsa-miR-669c	
hsa-miR-29b-2	hsa-miR-27b	hsa-miR-302a	hsa-miR-376b	hsa-miR-513b	hsa-miR-1246	hsa-miR-3	hsa-miR-4	
hsa-miR-103-2	hsa-miR-30b	hsa-miR-101-2	hsa-miR-483	hsa-let-7a-5p	hsa-miR-1825	hsa-miR-602	hsa-miR-85	
hsa-miR-103a-2	hsa-miR-122	hsa-miR-34b	hsa-miR-485	hsa-let-7f-5p	hsa-miR-544a	hsa-miR-520b	hsa-miR-5703	
hsa-miR-103-1	hsa-miR-124-1	hsa-miR-34c	hsa-miR-488	hsa-miR-16-5p	hsa-miR-885	hsa-miR-9500	hsa-miR-5120	
hsa-miR-103a-1	hsa-miR-124-2	hsa-miR-299	hsa-miR-490	hsa-miR-17-5p	hsa-miR-1207	hsa-miR-1182	hsa-miR-6881	
hsa-miR-106a	hsa-miR-124-3	hsa-miR-301a	hsa-miR-491	hsa-miR-17	hsa-miR-1301	hsa-miR-4284	hsa-miR-7240	
hsa-miR-16-2	hsa-miR-125b-1	hsa-miR-99b	hsa-miR-146b	hsa-miR-17-3p	hsa-miR-29	hsa-miR-875	hsa-miR-1261	
hsa-miR-192	hsa-miR-128-1	hsa-miR-296	hsa-miR-202	hsa-miR-19b-3p	hsa-miR-4739	hsa-miR-3666	hsa-miR-295	
hsa-miR-197	hsa-miR-130a	hsa-miR-130b	hsa-miR-493	hsa-miR-24-3p	hsa-let-7	hsa-miR-513a	hsa-miR-4481	
hsa-miR-199a-1	hsa-miR-132	hsa-miR-30e	hsa-miR-432	hsa-miR-26a-5p	hsa-miR-148	hsa-miR-1268a	hsa-miR-1968	
hsa-miR-208a	hsa-miR-133a-1	hsa-miR-26a-2	hsa-miR-494	hsa-miR-32-5p	hsa-miR-216b	hsa-miR-619	hsa-miR-4791	

Appendix F

**Table 10:** microRNAs within the HCC biomarkers network. afp: alpha fetoprotein; f2/DCP/PIVKA II: des γ carboxyprothrombin; gpc3: glypican 3; spp1: secreted phosphoprotein 1 (osteopontin (OPN)); golm1: Golgi protein-73 (GP73); dkk1: Dickkopf-related protein 1; HCC: hepatocellular carcinoma

afp	f2	gpc3	spp1	golm1	golm1	dkk1	dkk1
hsa-miR-217-3p	hsa-miR-450a-1-3p	hsa-miR-3613-3p	hsa-miR-4753-3p	hsa-miR-27b-3p	hsa-miR-3681-3p	hsa-miR-493-3p	hsa-miR-519a-5p
hsa-miR-1285-5p	hsa-miR-218-1-3p	hsa-miR-1255b-2-3p	hsa-miR-181a-5p	hsa-miR-27a-3p	hsa-miR-6805-3p	hsa-miR-433-3p	hsa-miR-519c-5p
hsa-miR-620	hsa-miR-8059	hsa-miR-5581-3p	hsa-miR-5003-5p	hsa-miR-9985	hsa-miR-320c	hsa-miR-3658	hsa-miR-518f-5p
hsa-miR-1270	hsa-miR-4471	hsa-miR-6792-3p	hsa-miR-181b-5p	hsa-miR-143-3p	hsa-miR-5691	hsa-miR-4511	hsa-miR-4662a-3p
hsa-miR-12131	hsa-miR-1292-5p	hsa-miR-4691-5p	hsa-miR-4262	hsa-miR-4770	hsa-miR-320b	hsa-miR-1227-3p	hsa-miR-3916
hsa-miR-4311		hsa-miR-96-5p	hsa-miR-181d-5p	hsa-miR-493-5p	hsa-miR-23c	hsa-miR-4503	hsa-miR-520c-5p
hsa-miR-4679		hsa-miR-204-5p	hsa-miR-181c-5p	hsa-miR-6088	hsa-miR-320d	hsa-miR-3177-5p	hsa-miR-3125
hsa-miR-1228-3p		hsa-miR-211-5p	hsa-miR-4659a-3p	hsa-miR-513a-5p	hsa-miR-320a-3p	hsa-miR-4699-3p	hsa-miR-526a-5p
hsa-miR-4496		hsa-miR-8080	hsa-miR-4659b-3p	hsa-miR-3685	hsa-miR-4429	hsa-miR-4282	hsa-miR-522-5p
hsa-miR-651-3p		hsa-miR-3121-3p	hsa-miR-7-1-3p	hsa-miR-4687-3p	hsa-miR-6884-5p	hsa-miR-126-5p	hsa-miR-519b-5p
hsa-miR-548t-5p		hsa-miR-12133	hsa-miR-7-2-3p	hsa-miR-1277-5p	hsa-miR-485-5p	hsa-miR-3065-3p	hsa-miR-3133
hsa-miR-548az-5p		hsa-miR-1271-5p	hsa-miR-130a-5p	hsa-miR-4328	hsa-miR-5011-5p	hsa-miR-934	hsa-miR-518d-5p
hsa-miR-513b-5p		hsa-miR-548x-5p	hsa-miR-4307	hsa-miR-155-3p	hsa-miR-548av-5p	hsa-miR-1912-5p	hsa-miR-518e-5p
hsa-miR-4261		hsa-miR-548aj-5p	hsa-miR-130b-5p	hsa-miR-4316	hsa-miR-449c-3p	hsa-miR-8485	hsa-miR-6859-5p
hsa-miR-1264		hsa-miR-548f-5p	hsa-miR-520e-5p	hsa-miR-3159	hsa-miR-548k	hsa-miR-6881-5p	hsa-miR-523-5p
hsa-miR-494-3p		hsa-miR-548g-5p	hsa-miR-12135	hsa-miR-6865-5p	hsa-miR-335-5p	hsa-miR-587	hsa-miR-3927-5p
hsa-miR-1236-3p		hsa-miR-548aw	hsa-miR-4251	hsa-miR-3177-5p	hsa-miR-651-5p	hsa-miR-217-5p	hsa-miR-509-5p
hsa-miR-342-3p		hsa-miR-138-1-3p	hsa-miR-4422	hsa-miR-6756-5p	hsa-miR-6735-3p	hsa-miR-362-5p	hsa-miR-509-3-5p
hsa-miR-935		hsa-miR-6883-5p	hsa-miR-3065-5p	hsa-miR-6766-5p	hsa-miR-3973	hsa-miR-500b-5p	hsa-miR-5584-5p
hsa-miR-147b-5p		hsa-miR-6872-3p	hsa-miR-2117	hsa-miR-6815-5p	hsa-miR-382-5p	hsa-miR-6890-5p	hsa-miR-643
		hsa-miR-149-3p	hsa-miR-539-5p	hsa-miR-5692a	hsa-miR-9500	hsa-miR-4804-3p	hsa-miR-488-3p
		hsa-miR-527	hsa-miR-34b-3p	hsa-miR-7702	hsa-miR-3925-3p	hsa-miR-195-3p	hsa-miR-548at-5p
		hsa-miR-6785-5p	hsa-miR-12123	hsa-miR-145-5p	hsa-miR-3611	hsa-miR-16-2-3p	hsa-miR-6792-5p
		hsa-miR-4728-5p	hsa-miR-545-5p	hsa-miR-5195-3p	hsa-miR-3941	hsa-miR-6807-3p	hsa-miR-3159
		hsa-miR-518a-5p	hsa-miR-3529-3p	hsa-miR-5197-3p	hsa-miR-4672	hsa-miR-1-3p	hsa-miR-32-3p
		hsa-miR-8069	hsa-miR-4482-3p	hsa-miR-508-5p	hsa-miR-4801	hsa-miR-144-3p	hsa-miR-517-5p
		hsa-miR-570-3p	hsa-miR-3180-5p	hsa-miR-30d-3p	hsa-miR-3145-5p	hsa-miR-101-3p	hsa-let-7c-3p
		hsa-miR-642b-5p	hsa-miR-6732-3p	hsa-miR-30e-3p	hsa-miR-342-3p	hsa-miR-205-3p	hsa-miR-508-5p
		hsa-miR-4717-3p	hsa-miR-513c-3p	hsa-miR-30a-3p	hsa-miR-589-3p	hsa-miR-206	hsa-miR-338-5p
		hsa-miR-3652	hsa-miR-518a-5p	hsa-miR-4782-3p	hsa-miR-451b	hsa-miR-501-5p	hsa-miR-186-5p
		hsa-miR-202-3p	hsa-miR-513a-3p	hsa-miR-6766-3p	hsa-miR-520g-3p	hsa-miR-579-3p	hsa-miR-302d-3p
		hsa-miR-4430	hsa-miR-527	hsa-miR-219a-5p	hsa-miR-520h	hsa-miR-664b-3p	hsa-miR-302b-3p
		hsa-miR-1468-3p	hsa-miR-577	hsa-miR-495-5p	hsa-miR-10395-3p	hsa-miR-302e	hsa-miR-5692a
		hsa-miR-450b-5p	hsa-miR-302f	hsa-miR-499a-3p	hsa-miR-7974	hsa-miR-219a-1-3p	hsa-miR-302c-3p
		hsa-miR-5681a	hsa-miR-548n	hsa-miR-499b-3p	hsa-miR-1251-3p	hsa-miR-4733-5p	hsa-miR-302a-3p
		hsa-miR-8055	hsa-miR-664a-3p	hsa-miR-219b-3p		hsa-miR-4639-3p	
		hsa-miR-4803	hsa-miR-6809-3p	hsa-miR-3140-3p		hsa-miR-140-3p	
		hsa-miR-4540	hsa-miR-3606-3p	hsa-miR-4276		hsa-miR-6505-5p	
		hsa-miR-140-5p	hsa-miR-3680-3p	hsa-miR-6733-5p		hsa-miR-3064-3p	
		hsa-miR-6842-3p	hsa-miR-6875-3p	hsa-miR-6739-5p		hsa-miR-613	
		hsa-miR-765	hsa-miR-153-5p	hsa-miR-3153		hsa-miR-103a-3p	
			hsa-miR-449b-3p	hsa-miR-1910-5p		hsa-miR-4727-5p	
			hsa-miR-525-5p	hsa-miR-216a-3p		hsa-miR-3974	
			hsa-miR-520a-5p	hsa-miR-128-3p		hsa-miR-107	
			hsa-miR-6885-3p	hsa-miR-8084		hsa-miR-3944-5p	
			hsa-miR-4786-3p	hsa-miR-6826-3p		hsa-miR-6738-3p	

Appendix G 

**Table 11:** Component scores for tumor-suppressive activity of microRNAs that are within the MASLD-HCC network AND the HCC biomarker network. spp1: secreted phosphoprotein 1 (osteopontin (OPN)); golm1: Golgi protein-73 (GP73); dkk1: Dickkopf-related protein 1; MASLD-HCC: metabolic dysfunction-associated steatotic liver disease-hepatocellular carcinoma

microRNA	Expression	Functional validation	Mechanistic clarity	Clinical correlation	Oncogenic safety	Total score	HCC biomarker gene
hsa-miR-577	2	1	2	3	3	11	spp1
hsa-miR-130b-5p	0	0	0	0	0	0	spp1
hsa-miR-9500	0	0	0	0	1	1	golm1
hsa-miR-101-3p	3	3	3	2	3	14	dkk1
hsa-miR-206	3	2	3	2	3	13	dkk1
hsa-miR-613	2	2	3	2	3	12	dkk1
hsa-miR-103a-3p	1	1	1	2	0	5	dkk1
hsa-miR-107	1	2	2	1	0	6	dkk1
hsa-miR-219a-1-3p	0	0	0	0	0	0	dkk1

Appendix H 

**Table 12:** Component scores for downregulated expression level of microRNAs that are within the MASLD-HCC network AND the HCC biomarker network AND are tumor-suppressive. dkk1: Dickkopf-related protein 1; MASLD-HCC: metabolic dysfunction-associated steatotic liver disease-hepatocellular carcinoma

microRNA	Expression	Sample size	Functional validation	Clinical correlation	Large dataset validation	Total score	HCC biomarker gene
hsa-miR-101-3p	27	19	20	13	15	94	dkk1
hsa-miR-206	27	19	20	9	15	90	dkk1
hsa-miR-613	27	14	20	9	15	85	dkk1
